# Commissioning of a 1.5T Elekta Unity MR‐linac: A single institution experience

**DOI:** 10.1002/acm2.12902

**Published:** 2020-05-20

**Authors:** Jeffrey E. Snyder, Joël St‐Aubin, Sridhar Yaddanapudi, Amanda Boczkowski, David A.P. Dunkerley, Stephen A. Graves, Daniel E. Hyer

**Affiliations:** ^1^ Department of Radiation Oncology University of Iowa Iowa City IA USA; ^2^ Department of Radiology University of Iowa Iowa City IA USA

**Keywords:** adaptive radiotherapy, commissioning, IMRT, MRI‐Linac, unity

## Abstract

MR image‐guided radiotherapy has the potential to improve patient care, but integration of an MRI scanner with a linear accelerator adds complexity to the commissioning process. This work describes a single institution experience of commissioning an Elekta Unity MR‐linac, including mechanical testing, MRI scanner commissioning, and dosimetric validation. Mechanical testing included multileaf collimator (MLC) positional accuracy, measurement of radiation isocenter diameter, and MR‐to‐MV coincidence. Key MRI tests included magnetic field homogeneity, geometric accuracy, image quality, and the accuracy of navigator‐triggered imaging for motion management. Dosimetric validation consisted of comparison between measured and calculated PDDs and profiles, IMRT measurements, and end‐to‐end testing. Multileaf collimator positional accuracy was within 1.0 mm, the measured radiation isocenter walkout was 0.20 mm, and the coincidence between MR and MV isocenter was 1.06 mm, which is accounted for in the treatment planning system (TPS). For a 350‐mm‐diameter spherical volume, the peak‐to‐peak deviation of the magnetic field homogeneity was 4.44 ppm and the geometric distortion was 0.8 mm. All image quality metrics were within ACR recommendations. Navigator‐triggered images showed a maximum deviation of 0.42, 0.75, and 3.0 mm in the target centroid location compared to the stationary target for a 20 mm motion at 10, 15, and 20 breaths per minute, respectively. TPS‐calculated PDDs and profiles showed excellent agreement with measurement. The gamma passing rate for IMRT plans was 98.4 ± 1.1% (3%/ 2 mm) and end‐to‐end testing of adapted plans showed agreement within 0.4% between ion‐chamber measurement and TPS calculation. All credentialing criteria were satisfied in an independent end‐to‐end test using an IROC MRgRT phantom.

## 1 Introduction

Magnetic resonance (MR)‐guided adaptive radiotherapy is being implemented in an increasing number of institutions worldwide. Magnetic resonance images provide superior soft tissue contrast compared to other image‐guided radiotherapy techniques such as kilovoltage cone‐beam computed tomography (kV‐CBCT). In addition to enhanced soft tissue contrast, MRI linear accelerators (MR‐linacs) allow for repeat imaging, near real‐time intrafraction imaging without additional radiation dose, and have the ability to perform target tracking and gated radiotherapy treatments.^1^, ^2^, ^3^, ^4^, ^5^ MR‐linacs also provide the added utility of being able to create adapted treatment plans to account for daily anatomical variations.^6^ Future work may allow for margin reduction and dose painting based on functional MR imaging.^7^

The Elekta (Stockholm, Sweden) Unity MR‐linac was approved for clinical use by the U.S. Food and Drug Administration (FDA) for patient treatments in 2018, making it the second commercially available MR‐linac along with the ViewRay MRIdian (ViewRay Inc., Oakwood, USA).^8^ The Unity couples a 1.5 Tesla Philips big‐bore MRI (Philips Healthcare, Amsterdam Netherlands) and a single‐energy 7 MV flattening filter‐free (FFF) standing‐wave linear accelerator.^9^ The radiation‐generation system uses a 160 leaf multileaf collimator (MLC) similar to the Agility MLC found on standard Elekta linacs. The maximum field size in the isocenter plane is 57.4 cm (crossplane) by 22.0 cm (inplane). Collimator rotation is disabled in this design, and the MLC leaves move in the superior/inferior direction with respect to patient anatomy with a leaf width of 7.175 mm at the isocenter plane.^10^ The Unity has a source to isocenter distance of 143.5 cm and an inner‐bore diameter of 70 cm. The system is currently only capable of delivering Intensity Modulated Radiotherapy (IMRT) and 3D conformal treatments.

While the Unity shows promise for improving patient care, it also poses significant commissioning challenges which are not present for standard linacs. Some of these challenges include performing reference dosimetry in nonstandard geometry,^11^ accounting for dosimetric effects from the Lorentz force on secondary electrons,^10^, ^12^, ^13^ and ensuring coincidence between the imaging and radiation coordinate systems. As the MRI is used directly for treatment planning in some adaptive workflows, the geometric accuracy, effect of the linac and gantry components on the MRI, and general image quality of the MRI requires evaluation. In addition to these challenges, all quality assurance equipment must be MR compatible, and the impact of the magnetic field on the response of the QA equipment including ion chambers must be well characterized.^14^, ^15^, ^16^, ^17^, ^18^ The characterization of a research version of the Monaco treatment planning system (TPS)^19^ and the commissioning of the MRI scanner using research MR sequences that are not widely available have been previously reported.^20^ Despite these initial efforts, there is little published guidance on commissioning the Elekta Unity in its current FDA‐approved state. The aim of this work is to describe the full commissioning process of the clinically approved Elekta Unity MR‐linac. Major components which will be discussed include mechanical testing, validation of the dosimetry system including end‐to‐end testing, and the initial commissioning of the MRI scanner using clinically available MRI sequences. Alternative measurement techniques other than described in this study may be utilized, but the main intent of this manuscript is to provide results from major commissioning tasks which can be used as a benchmark for other centers commissioning Unity MR‐linacs.

## 2 Methods

### 2.A Mechanicals

#### 2.A.1 MV isocenter

Radiation isocenter walkout as a function of gantry angle on the Elekta Unity is most readily evaluated using a Winston‐Lutz test. In general terms, this test involves placing a small radio‐opaque marker at isocenter and acquiring images from a number of gantry angles. The relative change in position of the marker with respect to the edges of the radiation field, as defined by the MLC, represents the radiation isocenter walkout. In this work, the Elekta MV Alignment Phantom was utilized which includes a central ball bearing (BB) that is visible using the MV portal imaging system. The phantom also includes two rings of smaller BBs in the superior/inferior direction which are used to check the rotation of the phantom. While not important for this test, the rotation of the phantom is important for calibration of the QA Platform, an adjustable jig that is calibrated to isocenter using the MV Alignment Phantom and subsequently used to position QA equipment in place of a field light or transverse laser.

**Fig. 1:**
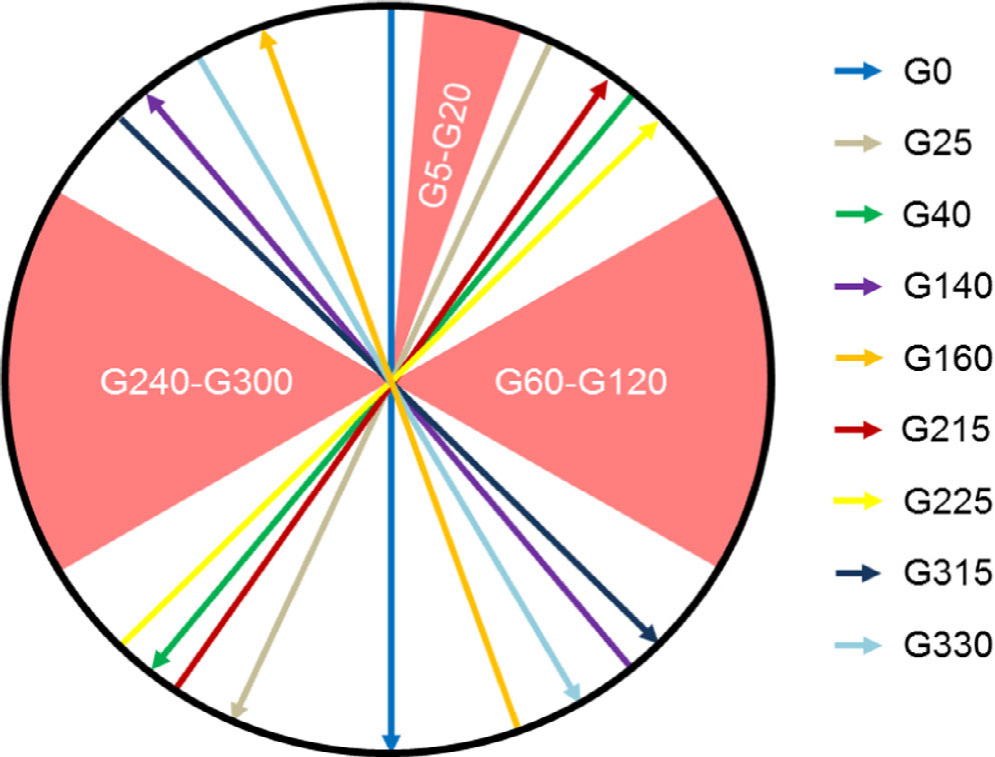
Beam angles used to evaluate radiation isocenter are shown. Gantry angles between 5˚ and 20˚ are avoided due to the cryostat pipe and the lateral gantry angles are excluded to avoid imaging the mounting stand for the MV alignment phantom which impacts the ability to reliably detect the field edges using RIT.

To evaluate the isocenter size, MV images were acquired at the angles shown in Fig. 1 using a 3 × 3 cm^2^ field. Images were analyzed using the RIT v6.7.64 (Radiological Imaging Technologies, Colorado Springs, CO) 3D Stereotactic Analysis application.

#### 2.A.2 MR‐MV isocenter coincidence

The MRI and rotating gantry which houses the linac are mechanically aligned during installation.

The goal of the installation process is to align the geometric center of the MRI to within 0.5 mm of the rotating gantry mechanical isocenter. After installation is complete, it is important to verify the coincidence between the MR and MV isocenter as any misalignment will result in systematic errors if not properly accounted for. This was done by using the Elekta MR‐to‐MV Alignment Phantom which contains seven ZrO_2_ spheres in a known geometry surrounded by plastic and a copper sulfate solution. These ceramic spheres appear as high‐density BBs on the MV portal imager and as signal voids on T1‐weighted MR images.

To perform this test, a series of 10 MV images were acquired at varying gantry angles (0, 60, 78, 102, 117, 180, 240, 258, 282, and 300) with a field size of 22 × 9 cm^2^. Without moving the phantom, a T1‐weighted MRI was also acquired. The images were then analyzed using the Elekta‐provided QA Alignment Software. This software is used to detect the center of each sphere in both the MR and MV images. The sphere centers are calculated using an intensity gradient for each voxel which partially intersects the sphere. A line is drawn along the gradient direction and the intensity of all gradient lines are added which results in a local maximum at the location of the sphere center. The QA alignment software then compares the position of the sphere centers between the two systems to determine the MR‐MV isocenter coincidence.

#### 2.A.3 MLC positional accuracy

**Fig. 2:**
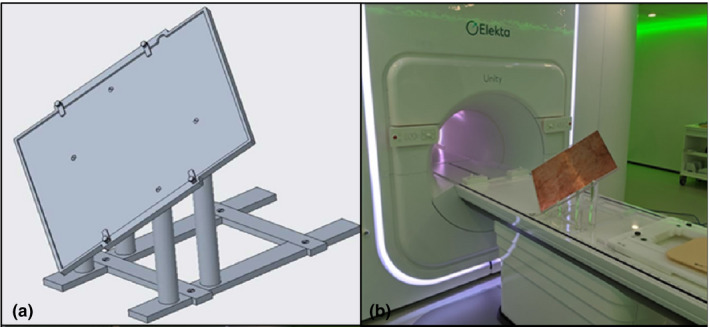
Design for prototype film positioning platform (left) and manufactured platform with film positioned between 2 mm copper sheets (right).

The design of the MLC on the Elekta Unity was adapted from traditional Elekta linear accelerators with only minor design changes.^21^ As such, positional accuracy of the system can be evaluated by means similar to any traditional linear accelerator with some additional limitations. Most commonly on a traditional linear accelerator, the MV imaging system is used to acquire a picket fence image which is used to evaluate the accuracy of the leaves. Unfortunately, due to the distance to the MV imaging panel (265.7 cm from the source) and its limited size (41 × 41 cm^2^ physical dimension with an imaging dimension of 22 × 9.5 cm^2^), only the central 30 leaf pairs can be captured using the MV imager. Therefore, in this work, a film‐based picket fence test was developed using an in‐house developed film platform [Fig. 2(a)] to capture the position of all 80 leaf pairs. Four, 8‐mm‐wide open strips were positioned such that a 0.2 cm gap was present between each strip, resulting in three underexposed strips centered at positions of −1, 0, and + 1 cm from the center of the MLC bank. A film platform was manufactured to hold a single piece of 32.5 × 43.2 cm^2^ EBT3‐1417 radiochromic film at 123.3 cm from the source with the film oriented with the exposure along the diagonal axis such that the entire radiation field fit onto a single film (Fig. 2). During irradiation, the film was positioned between 0.22‐cm‐thick pieces of copper to provide buildup and mitigate the electron return effect. The film was digitized using an Epson 12000XL scanner at 75 DPI and processing was automated using in‐house developed software using MATLAB (Mathworks, Natick, MA) to identify each leaf position and the location of the 0.2 m underexposed strips from the resulting profiles.

#### 2.A.4 MLC transmission

The Elekta MLC leaves are made of a tungsten nickel iron alloy (5% Ni‐Fe) with a height of 9 cm. The sides of each leaf are flat but the entire bank of leaves is defocused from the x‐ray source to reduce interleaf transmission. Evaluation of MLC leakage was performed by delivering 2000 MU with both an open 10 × 10 cm^2^ field and an MLC blocked field and comparing the charge reading from an ion chamber in each setting. The measurement was also compared to the same setup in the treatment planning system.

### 2.B MRI commissioning

#### 2.B.1 Magnetic field homogeneity

The magnetic field homogeneity of the Philips Marlin MRI was determined by examining the B_0_ map over a 350‐mm‐diameter spherical volume (DSV). A 400‐mm‐diameter Philips body phantom was used in the generation of the B_0_ maps, oriented in the transverse, sagittal, and coronal planes of the MRI. A dual‐echo method was used to obtain the B_0_ field map using a repetition time (TR) of 65 ms, echo times of $TE_{1}=3.51ms$ and $TE_{2}=6.80ms$ to minimize phase wrapping, a flip angle of 20°, and using a slice resolution of 5 mm and an in‐plane resolution of 2 × 2 mm.^2^, ^22^, ^23^, ^24^ The B_0_ map was created from the phase data of both echoes using,

(1) $$\Delta B_{0}=\frac{\Delta \phi }{\gamma \left(TE_{2}-TE_{1}\right)}$$

where Δϕ is the phase difference between the unwrapped phase images, and γ=42.58MHz/T is the gyromagnetic ratio for protons in a 1.5 T magnetic field. Phase unwrapping was performed using the freely available MEDI Toolbox from Cornell University (http://pre.weill.cornell.edu/mri/pages/qsm.html),^25^ and no phase unwrapping errors were detected in the regions of interest. Due to the fact that the linac gantry rotates around the patient during treatment as the MR acquires images, the quality of the magnetic decoupling of the linac from the MRI was assessed via B_0_ maps generated at gantry angles of 30, 60, 90, 120, 180, 210, 270, 300, and 330° with the Philips body phantom oriented in the transverse plane. The B_0_ maps at each angle were compared to the B_0_ map at gantry angle 0° to find the maximum change in homogeneity relative to gantry angle 0°.

#### 2.B.2 Geometric accuracy

**Fig. 3:**
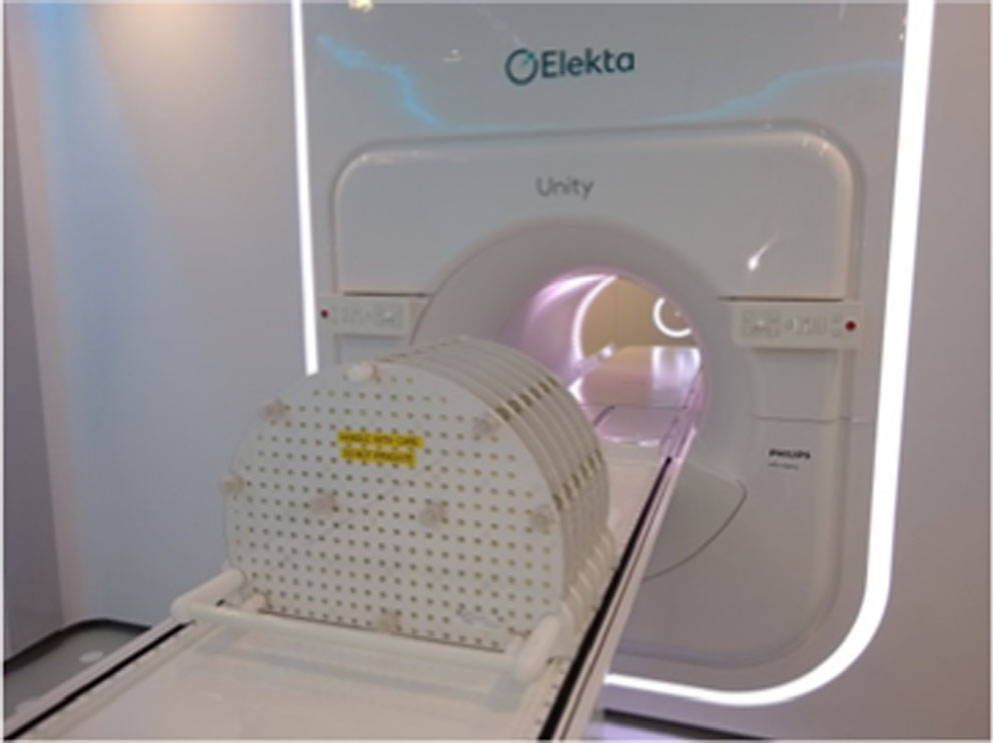
Geometric distortion phantom supplied with the Elekta Unity MRI‐linac.

Geometric accuracy of the MRI was determined using the vendor supplied 3D geometry phantom with dimensions of 500 × 375×330 mm^3^ (Fig. 3). The markers are situated in seven planes separated by 55 mm and are spaced 25 mm apart within the plane. The position of the markers of the 3D distortion phantom, determined using vendor supplied software, was compared with the expected positions which are determined based on the manufacturing of the phantom to produce a distortion map. The vendor software masks out the background for each marker separately and determines the centroid and radius of each marker in the masked image. Philips states an accuracy of 0.2 mm or less in the detection of each marker.

#### 2.B.3 Gradient fidelity

Gradient errors were determined using the vendor supplied 3D geometry phantom and vendor analysis software to determine the expected and measured locations of the markers (see geometric accuracy 2.B.2). To determine the gradient linearity, the geometric accuracy test was performed twice: once with a positive readout gradient and again with a negative readout gradient. Based on the expected and measured locations of the markers, gradient linearity can be determined by following the procedure described by Baldwin et al.^26^

#### 2.B.4 RF interference

Due to the close proximity of radiofrequency (RF) power during linac operation, assessing RF interference, which presents as noise in MRI, is critical. RF interference was determined by placing a long conducting wire within the MRI (to increase sensitivity) and assessing the noise in the images for the following conditions: a) linac off, b) magnetron powered up with no radiation, c) MLC moving with no radiation, and d) radiation on. A comparison between these conditions was performed using the vendor supplied automatic window/level feature on the Marlin software. The vendor specifies that the automatic window/level should be around 1200/700 which indicates acceptable noise. Any major deviation from these values indicates the presence of spurious noise.

#### 2.B.5 Image quality

Standard image quality tests were performed using the American College of Radiology (ACR) large phantom. Specifically, high‐contrast resolution, slice thickness accuracy, slice position accuracy, percent integral uniformity, percent signal ghosting, and low‐contrast object detectability were assessed using the ACR criteria. T1‐weighted images were acquired with TE = 20 ms, TR = 500 ms, and a flip angle of 90°. The T2‐weighted images were acquired using TE = 20 ms, TR = 2000 ms, and a flip angle of 90°. The images were analyzed using the RIT v6.7.64 MRI ACR module.

#### 2.B.6 Triggered imaging accuracy

For abdominal tumors that move (e.g., liver, pancreas), a T2‐weighted navigator‐triggered imaging protocol is available on the Marlin. The system uses a navigator‐triggered method to acquire the 3D images at the end‐exhalation breathing phase. The accuracy of the T2‐weighted triggered imaging sequence acquired at the end‐expiration phase was assessed using the Quasar MRI 4D motion phantom (Modus Medical Devices, London ON). The MRI 4D phantom contains separate compartments including a spherical target within a moving cylinder. The image of the spherical phantom target was automatically contoured in Velocity AI (Varian Medical Systems, Palo Alto CA) with a constant window/ level of 2000/ 1000 and a contouring threshold of 1050. Accuracy of the triggered images was determined for a 20 mm periodic sinusoidal motion at breathing rates of 10, 15, and 20 breaths per minute (bpm), and for an irregular breathing pattern where throughout the acquisition the breathing amplitude was varied between 18.5 and 21.4 mm, the breathing rate was varied between 10 and 20 bpm, and the form of the wave function was varied among sin,^2^ sin,^4^ and sin.^6^ The contoured target centroid positions together with the contoured volumes for the tests with motion were compared with the centroid position and volume of the stationary target.

### 2.C Dosimetric validation

#### 2.C.1 Reference dosimetry calibration

Reference dosimetry calibration was carried out using an MRI compatible 1D water tank (PTW, Freiburg, Germany) and a PTW farmer‐type ionization chamber (TN30013) with a valid ADCL calibration. This process was done following the guidelines provided by O’Brien et al.^11^ including the use of a chamber‐specific $k_{B}$ factor which accounts for changes in chamber response due to the presence of the magnetic field. Briefly, measurements were performed with the chamber aligned parallel with the magnetic field and at a gantry angle of 90˚ which removes output dependence due to varying helium levels. The previously reported $k_{B}$ factor for this chamber used in the parallel orientation with respect to the magnetic field is 0.994.^11^ All measurement depths accounted for the water equivalent thickness of the acrylic 1D tank wall. The absolute positioning of the ion chamber at isocenter was validated through the acquisition of orthogonal MV EPID images. Due to the extended SSD of the Unity, the beam quality specifier measured was $TPR_{10}^{20}$ .^27^ The $TPR_{10}^{20}$ was then converted into a %dd(10)_x_ using the formalism provided by Kalach et al.^28^ The remainder of the reference dosimetry calibration procedure followed the recommendations provided by AAPM Task Group Report 51 addendum.^29^ The Unity is designed to have a fixed‐dose rate of 425 MU/min at the point of calibration (1 cGy/MU) regardless of what depth that calibration is performed at. In this work, a calibration depth of 10 cm was chosen instead of *d*_max_ in order to maximize the delivered dose rate. Calibration was performed with a source‐to‐axis distance (SAD) setup which consisted of an SSD of 133.5 cm and a depth of 10 cm. Thermoluminescent dosimeters (TLD) provided by the University of Wisconsin Radiation Calibration Laboratory were irradiated on the Unity with 100 cGy and then read at the calibration laboratory as an independent validation of the clinical reference dosimetry performed. The TLDs were housed in a cylindrical solid water holder with minimal air gaps which fit into the ion‐chamber holder of the 1D water tank. The cylindrical holder can be imaged without the TLDs in place to assist with positioning prior to irradiation.

#### 2.C.2 Beam characterization—PDD and profiles

All of the measurements for beam characterization were acquired utilizing an Elekta‐provided proprietary MR‐safe 3D water tank which has dimensions of 62.8 × 44.2 × 24.1 cm^3^. The PDDs and profiles were acquired as per the requirements of the Monaco treatment planning system and recommendations provided by Elekta. Due to the limitations of the Unity bore and the water tank dimensions, a maximum scan depth of 12 cm was achievable when the gantry was set to 0º. With the gantry at 270º or 90º, the maximum scan depth was 38 cm, but the field size was limited. Hence, PDDs and profiles for field sizes of 2 × 2, 3 × 3, 5 × 5, 10 × 10, 15 × 15, 22 × 22, 40 × 22, 53.5 × 22, and 57.4 × 22 cm^2^ were acquired at depths of 1.3 cm, 5 cm, and 10 cm with the gantry set to 0º, and field sizes of 2 × 2, 3 × 3, 5 × 5, 10 × 10, and 16 × 16 cm^2^ were acquired at depths of 1.3 cm, 5 cm, 10 cm, 20 cm, and 30 cm with the gantry set to 270º. A maximum depth of 12 cm and 35 cm at gantry position 0º and 270º, respectively, was achievable for the PDD measurements. A PTW 0.07 cc ionization chamber (TN31021) and a PTW microdiamond detector (TN60019) were used for all measurements. The raw profile scans were processed using the PTW MEDPHYSTO software. Symmetry was defined within the central 80% of the full width at half maximum of the processed profile. Beam flatness was evaluated by examining the relative percent dose at specified off‐axis distances and comparing to vendor specifications. Measured PDDs and profiles were compared to the TPS calculation using a gamma analysis of 2% dose difference (DD) and 2 mm distance to agreement (DTA) with global normalization.

#### 2.C.3 Cryostat characterization and output versus gantry

The cryostat is an insulated container that maintains cryogenic temperatures using liquid helium to achieve superconductivity of the MRI. The construction of the cryostat is not entirely uniform over the surface of the magnet resulting in differences in the beam attenuation. As part of the commissioning process, it is necessary to characterize these attenuative properties which required the removal of the posterior coils and bridge from the couch so they do not affect the measurements. Attenuation measurements were made following the methodology described by Woodings et al.^30^ Briefly, an ionization chamber in a build‐up cap was placed such that the chamber center was at the MV isocenter. MV images, acquired on the EPID at the four cardinal gantry angles (0º, 90º, 180º, and 270º), were analyzed using the Elekta provided cryostat characterization alignment tool to ensure that the chamber was positioned at the isocenter of the machine. After alignment of the chamber, the output for a 10 × 10 cm^2^ field size every 2º was measured. This angle‐dependent transmission map was used to model the cryostat in the Monaco treatment planning system. To validate the implementation of this transmission map in the TPS, a plan was created in Monaco to mirror the experimental setup.

In addition to the cryostat, output versus gantry measurements are also strongly influenced by the presence of the MR‐linac couch. In order to measure the change in output versus the gantry angle in the presence of the couch, the ArcCheck (Sun Nuclear, Melbourne FL) device was setup on the calibrated QA platform provided by Elekta. The ionization chamber was positioned at the center of the ArcCheck and charge readings were obtained for a 10 × 10 cm^2^ field size at different gantry angles. The individual ionization chamber readings were normalized to the 90º gantry angle.

#### 2.C.4 IMRT measurements

The commissioning of IMRT treatments was performed using datasets provided by AAPM TG‐119^31^ and AAPM Medical Physics Practice Guideline (MPPG) 5.a.^32^ These plans would serve as reference plans in the clinical workflow. In total, 11 reference datasets were measured. Each of these reference datasets was calculated with a 2 mm dose grid and a 1% statistical uncertainty per calculation.

In addition to the above tests, the online adaptive functionality and dosimetric accuracy of the TPS were also tested. A treatment plan was created on a head and neck CT scan with a registered secondary T1 MRI dataset (reference plan). The MRI dataset was then deformed using ImSim QA software (Oncology System Limited, UK)^33^ and imported into the TPS representing a pretreatment MRI that would be acquired on the Unity. All possible online adaption methods for the Unity have been previously described in the literature.^6^ For this testing, two different plan adaptions methods were used to create IMRT plans including the adapt‐to‐position (ATP) method and the adapt‐to‐shape (ATS) method.^6^ In the ATP methodology, the optimize weights option was chosen to recreate the dose volume histogram of the reference CT plan. The ATS method allows recontouring of all OARs and the target structures, and then performs a full‐dose re‐optimization on the daily MRI using bulk electron density assignments. In the ATS workflow, re‐optimization was performed from fluence rather than using previously calculated segments as a starting point. The head and neck reference plan and both adapted plans were calculated using a 3 mm dose grid and 1% statistical uncertainty per calculation.

All test measurements were performed using the ArcCheck, which was placed onto the calibrated QA platform aligned to isocenter. Gamma analysis was performed in accordance with AAPM TG‐218,^34^ specifically at 3% DD and 2 mm DTA, and global normalization with a 10% low‐dose threshold applied. In addition to gamma analysis, point‐dose measurements for the adaptive workflow test cases were also measured.

A CT scan of the ArcCheck in the QA platform was acquired and imported into the Monaco TPS. The ArcCheck and QA platform were contoured to apply electron density (ED) overrides. The QA platform was assigned a relative ED of 1.2 based on recommendations from Elekta. Setting the electron density of the ArcCheck for use with the Monaco TPS has been previously reported.^35^

#### 2.C.5 End‐to‐end testing

End‐to‐end tests were performed during commissioning using a CIRS thorax phantom (model 008Z CIRS, Norfolk, VA) composed of heterogenous materials and a 0.07 cc ion‐chamber insert. An additional end‐to‐end test was done using the MRgRT Head and Neck phantom provided by IROC. The irradiation of this phantom and subsequent analysis by IROC serves as an independent validation of the end‐to‐end process.

The CIRS phantom consists of a several OARS of varying electron densities as measured on our CT scanner including lungs (0.082ED), kidneys (1.058ED), liver (1.071ED), bone (1.151ED), and spinal cord (1.064ED). The respiratory motion component of this phantom was not used for end‐to‐end testing. The phantom was scanned using a Siemens Biograph PET/CT scanner with 2 mm slice thickness. An Elekta provided CT table overlay identical to the Unity treatment couch was placed on the CT scanner prior to image acquisition. Images were transferred to the Monaco TPS for treatment plan generation. The treatment plan utilized five steps and shoot IMRT fields and was prescribed to a dose of 6300 cGy in 35 fractions. The dose calculation was performed with a 3 mm dose grid and a 1% statistical uncertainty per plan. The phantom was positioned on the treatment table and a 2‐minute T2‐weighted pretreatment image was acquired and registered to the reference CT plan. One ATP plan using the optimize weights option and one ATS plan was created as described in section 2.C.4. A 0.07 cc ion‐chamber, cross‐calibrated against a farmer‐type ion chamber with an ADCL calibration, was inserted into the phantom and the measured dose was recorded for each delivered plan adaptation method. A small amount of water was added to the milled ion‐chamber insert with the intention of minimizing air gaps between the chamber and phantom. The measured chamber dose was compared against the TPS‐reported point‐dose value.

The MRgRT Head and Neck phantom from IROC contains two PTV’s and a spinal cord as the primary OAR. An axial and sagittal plane GafChromic film are located in the high‐dose PTV as well as eight separate TLD locations. A CT scan was acquired and transferred to Monaco as previously described. The plan was created using a single‐fraction simultaneous integrated boost with one PTV (0.845ED) receiving 660 cGy and the second PTV (0.845ED) receiving 540 cGy. The spinal cord (1.080ED) max‐dose constraint was 450 cGy. The plan was created using 11 IMRT fields and a 2 mm dose grid with 1.0% statistical uncertainty per calculation. A 2 mm dose grid was chosen in accordance with AAPM TG 101 guidelines.^36^ The pretreatment image was a 2‐minute 3D T1‐weighted sequence and the ATS option was used for adaptive plan calculation. The phantom was irradiated and sent to IROC for analysis. TLD measurements were compared to reported TPS values and the film planes were analyzed using a 7% DD and 4 mm DTA criteria as specified by IROC credentialing standards.

## 3 Results

### 3.A Mechanical testing

#### 3.A.1 MV isocenter

The Elekta Unity isocenter walkout was minimal due to the rigidity of the ring‐mounted linear accelerator. The standard deviation of ball‐field displacements from all gantry angles evaluated was 0.12, 0.07, and 0.14 mm on the X (left/right), Y (sup/inf), and Z (ant/post) axis, respectively.

#### 3.A.2 MR‐MV isocenter coincidence

A baseline isocenter coincidence between the MRI and MV system was found to be 0.307, 0.998, and −0.015 mm in the X, Y, and Z directions, respectively. This transform is applied in the treatment planning system each time Unity MR images are imported.

#### 3.A.3 MLC positional accuracy

**Fig. 4:**
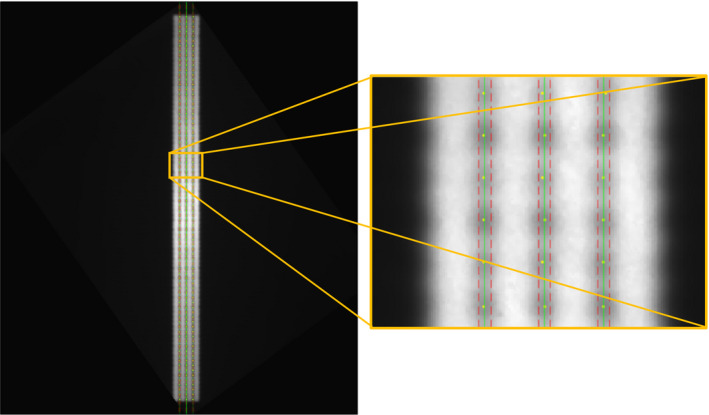
Digitized film overlaid with localized leaf positions (yellow points), expected centerline (green line), and positional tolerance (red line) for the full film (left) and a magnified view (right).

A quantitative analysis of the film‐based picket fence was performed on the scanned film shown in Fig. 4. Deviations from expected centerline leaf locations were within 0.5 mm for all 80 leaf pairs with an average and standard deviation of 0.1 ± 0.19 mm for all three strips.

#### 3.A.4 MLC transmission

The MLC transmission for the Y1 and Y2 leaf banks was measured as 0.67% and 0.72%, respectively. The leakage reported by the treatment planning system with the same setup was 0.75% and 0.71% for the Y1 and Y2 leaf banks, respectively, showing excellent agreement with measurement.

### 3.B MRI commissioning

#### 3.B.1 Magnetic field homogeneity

**Fig. 5:**
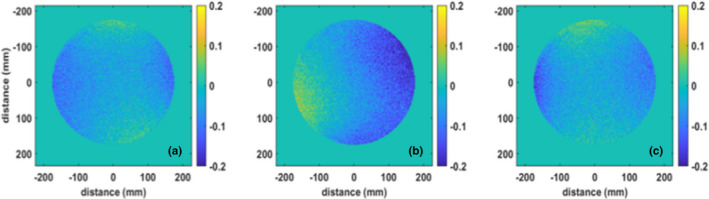
Parts per million (ppm) differences in magnetic field homogeneity among a) gantry 90—gantry 0, b) gantry 180—gantry 0, and c) gantry 270—gantry 0 over a 350‐mm‐diameter spherical volume.

The magnetic field homogeneity was determined over 350‐, 300‐, 200‐, and 100‐mm‐diameter spherical volumes (DSV) for acquisitions of the Philips body phantom in the transverse, coronal, and sagittal phantom orientations (Table 1). The maximum peak‐to‐peak variation with the body phantom in the transverse orientation was 3.91 ppm over a 350 mm DSV for all gantry angles tested. In addition, the root‐mean‐square difference of the magnetic field homogeneity over a 350 mm DSV between all gantry angles tested and gantry angle 0° was found to be 0.l0 ppm. Figure 5 shows B_0_ field difference maps comparing the magnetic field homogeneity for the cardinal gantry angles (90, 180, and 270) with gantry angle 0° for the 350 mm DSV.

**Table 1:** Magnetic Field homogeneity over 350‐, 300‐, 200‐, and 100‐mm‐diameter spherical volumes is presented for the Philips Body cylinder phantom in transverse, coronal, and sagittal orientations with the gantry angle at 0°.

	Peak‐to‐Peak Magnetic Field Homogeneity (ppm)			
	350 mm DSV	300 mm DSV	200 mm DSV	100 mm DSV
Transverse	3.62	2.56	1.56	0.71
Coronal	4.09	2.20	0.81	0.41
Sagittal	4.44	2.38	0.79	0.40

#### 3.B.2 Geometric accuracy

The geometric accuracy was assessed over 400, 350, 300, 200, and 100 mm DSVs. Table 2 gives the maximum distortions for the right‐left (RL), anterior‐posterior (AP), and superior‐inferior (SI) directions as well as the total distortion.

**Table 2:** Geometric distortion values in the right‐left (RL), anterior‐posterior (AP), and foot‐head (FH) directions, along with the maximum total distortion over a range of diameter spherical volumes are given.

	Maximum distortion (mm)				
	400 mm DSV	350 mm DSV	300 mm DSV	200 mm DSV	100 mm DSV
Total	1.1	0.8	0.7	0.4	0.2
RL	0.8	0.5	0.4	0.2	0.1
AP	0.9	0.7	0.6	0.4	0.2
FH	0.8	0.7	0.5	0.3	0.2

#### 3.B.3 Gradient fidelity

The linearity of each gradient is critical in reducing the geometric distortion. Following the procedure of Baldwin et al.,^26^ the geometric fidelity of the Marlin system was evaluated. Table 3 provides the maximum absolute error associated with gradient nonlinearity for the RL (x gradient), AP (y gradient), and SI (z gradient) directions for a range of DSVs.

**Table 3:** Geometric errors associated with gradient nonlinearity for each gradient is given for a range of diameter spherical volumes.

	Maximum distortion (mm)				
	400 mm DSV	350 mm DSV	300 mm DSV	200 mm DSV	100 mm DSV
RL (x gradient)	0.8	0.5	0.4	0.2	0.1
AP (y gradient)	0.7	0.6	0.4	0.3	0.1
FH (z gradient)	0.8	0.7	0.5	0.3	0.2

#### 3.B.4 RF interference

Using the Philips provided procedure of evaluating the noise in the images using the automatic window/level settings of the Marlin software, the impact of various linac components on the MRI was determined. Automatic window/level settings were determined for the various scenarios including when the linac is off (window (W) = 1268, level (L) = 730), the magnetron is energized, but the linac is not producing radiation (W = 1288, L = 741), only the MLCs are moving (W = 1183, L = 680), and when the radiation is on (W = 1290, L = 742). The Philips acceptance criteria is that no visible artifacts are seen in the images, and the window/level settings are around 1200/700.

#### 3.B.5 Image quality

Image quality was assessed using the ACR large phantom for T1‐ and T2‐weighted images produced on the Marlin. The results of the image quality tests are given in Table 4.

**Table 4:** ACR test acceptance criteria and measured results for T1‐ and T2‐weighted images on the Marlin.

	Acceptable result	Measured T1	Measured T2
High‐contrast resolution (slice 1)	≤ 1.0	1.0	1.0
Slice thickness accuracy (slice 1)	5.0±0.7mm	4.61	4.90
Slice position accuracy (slice 1)	-5mm<x<5mm	−0.82	‐1.06
Percent signal ghosting (slice 7)	≤ 0.025	0.0031	0.0009
Percent Integral Uniformity (slice 7)	≥ 87.5	91.81	91.91
Low‐contrast object detectability (slices 8–11)	> 9 spokes	15	15

#### 3.B.6 Triggered imaging accuracy

**Fig. 6:**
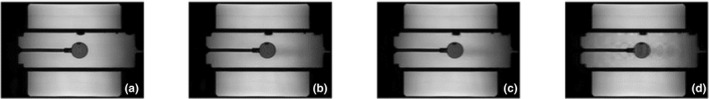
Quasar MRI 4D phantom images for a) no motion, and for a 20 mm periodic sinusoidal motion at b) 10 bpm, c) 15 bpm, and d) 20 bpm.

The triggering phase was validated to be at the end‐expiration phase of the breathing cycle for both head‐first and feet‐first orientations using the Quasar Modus 4D MRI phantom. The calculated centroid of the target for the triggered images differed from the stationary phantom image by 0.42, 0.75, and 3.0 mm for the periodic sinusoidal pattern for breathing rates of 10, 15, and 20 bpm, respectively, and by 0.45 mm for the irregular breathing pattern. The target contour volume for the no‐motion scan was determined to be 20.52 cm^3^, while the volumes for the periodic sinusoidal motion at 10, 15, and 20 bpm were found to be 20.87, 20.82, and 20.67 cm^3^, respectively, while the target volume was determined to be 20.43 cm^3^ for the irregular breathing pattern. Due to the spherical volume of the target, this represents an error of 0.19, 0.16, and 0.08 mm in the calculated diameter of the target for 10, 15, and 20 bpm for a periodic 20 mm sinusoidal motion, and −0.05 mm for the irregular breathing pattern. In addition to the centroid and volume impact of the triggered images, the motion artifacts were seen to increase with increasing breathing rate, as seen in Fig. 6.

### 3.C Dosimetric validation

#### 3.C.1 Reference dosimetry calibration

The $TPR_{10}^{20}$ beam quality specifier value measured was 0.704. Using the formalism provided by Kalach et al.^28^ this equates to a %*dd*(10)_x_ value of 70.24% and from the AAPM TG‐51 addendum^29^ a *k*_Q_ value of 0.986. TLDs were irradiated with an institution reported value of 100 cGy and were independently read out by an accredited dosimetry laboratory that reported a measured reading of 99.1 cGy ± 5%. The difference between the reported delivered dose and the independently measured dose was −0.9%.

#### 3.C.2 Beam Characterization—Pdd And Profiles

**Fig. 7:**
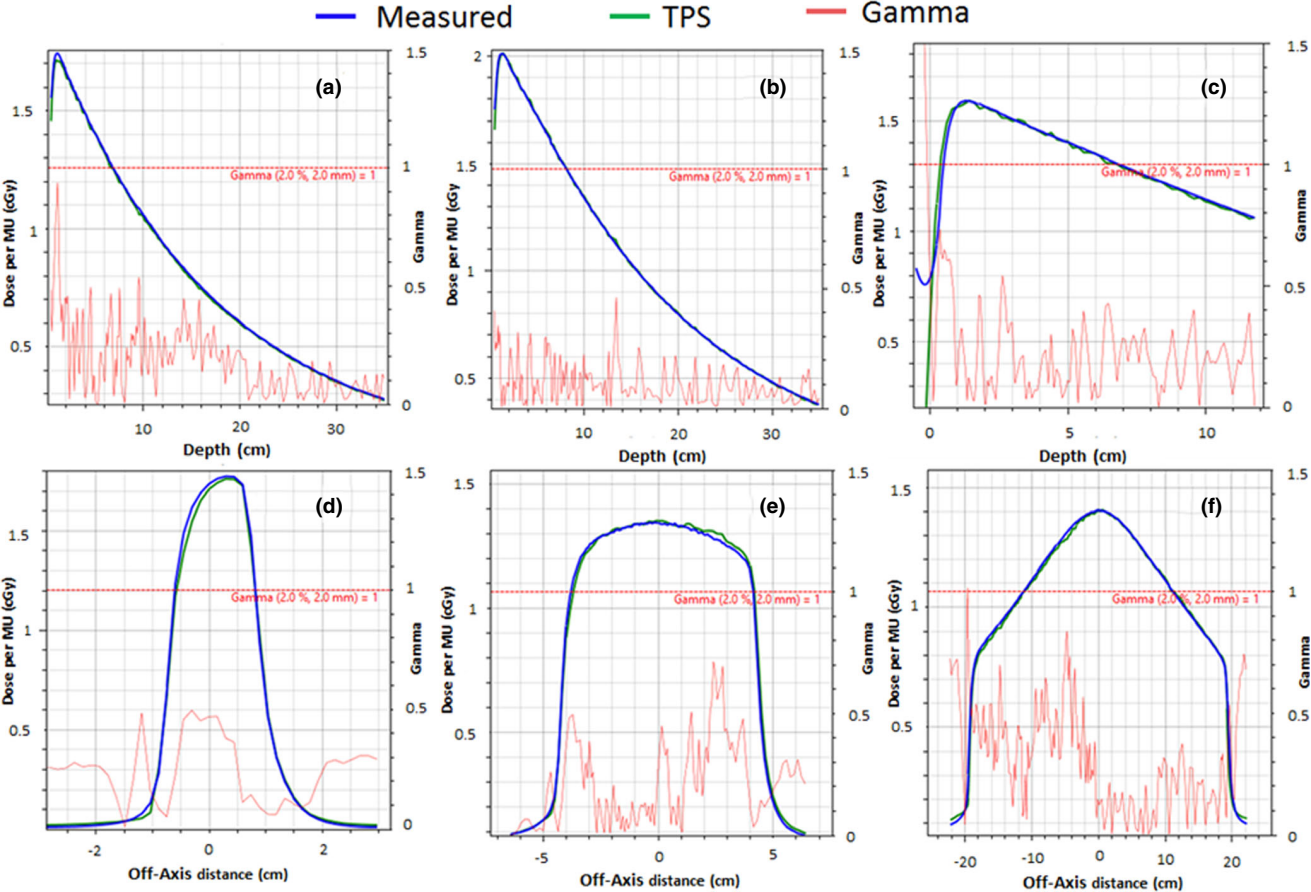
Gamma Analysis measured and TPS‐calculated PDDs and Profiles. a) 2 × 2 cm^2^ PDD at gantry 270˚ and 113.1 cm SSD. b) 10 × 10 cm^2^ PDD at gantry 270˚ and 113.1 cm SSD. c) 40 × 22 cm^2^ PDD at gantry 0˚ and 133.5 cm. d) 2 × 2 cm^2^ cross‐plane profile at 1.3 cm depth and gantry 270˚. e) 10 × 10 cm^2^ cross‐plane profile at 10 cm depth and gantry 270˚. f) 40 × 22 cm^2^ cross‐plane profile at 5 cm depth and gantry 0˚.

Figure 7 shows representative PDDs and profiles measured during commissioning. The measured PDDs and profiles were compared against the beam model, and the average gamma passing rate (2%/2 mm) was 99.7 ± 1.0%. The minimum gamma passing rate for any individual PDD or profile was 95.0%. In general, gamma values above 1 were only seen in the tail region of the profile and at the surface of the PDD for the largest measured field sizes.

#### 3.C.3 Cryostat characterization and output versus gantry

**Fig. 8:**
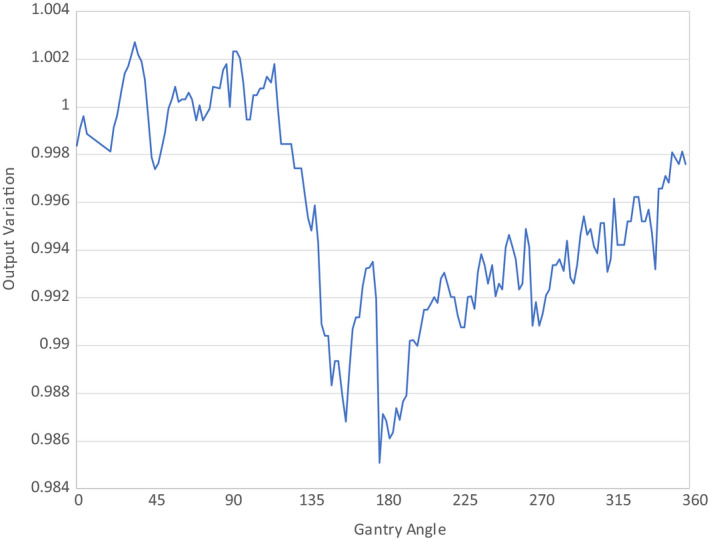
Angle‐Dependent Cryostat Attenuation Map. All results are normalized to the results measured at a gantry angle of 90°.

It was determined that the ionization chamber with the build‐up cap was positioned to be within ± 0.1 mm from the isocenter for the measurements based on the MV image analysis. With this setup, the angle‐dependent cryostat attenuation map was measured with results shown in Fig. 8.

The comparison between the measured cryostat attenuation and calculation showed agreement within 1.2% for all gantry angles. The addition of the MR‐linac couch into the output versus gantry measurements is shown in Table 5. Beams passing through the couch can be attenuated by as much as 16.5%. Elekta provides a couch model within Monaco TPS to account for these angle‐dependent variations. Using the couch model in the Monaco TPS, the discrepancy between measurement and calculation was as much as 2.76%. This discrepancy may be due in part to the steep attenuation gradient of the couch and partial volume effects of the ion chamber.

**Table 5:** Output versus Gantry with Incorporation of MR‐Linac Couch.

Gantry Angle (˚)	Monaco Calculation	Measurement	Difference (%)
0	1.011	0.996	1.49
45	1.002	0.989	1.31
90	1.000	1.000	0.00
135	0.820	0.835	−1.81
180	0.887	0.903	−1.79
225	0.821	0.844	−2.76
270	1.006	1.000	0.60
315	1.001	0.995	0.60

#### 3.C.4 IMRT measurements

The passing rates of all reference IMRT plans were greater than 96.7% (average = 98.3 ± 1.1%). The gamma passing rates for the adaptive head and neck plans were 99.6% and 99.7% for the adapt‐to‐position and adapt‐to‐shape plans, respectively. The online dose recalculation time for the adapt to position plan was 171 sec and 460 sec for the adapt to shape plan. The head and neck reference plan gamma pass rate was 99.2%. The point‐dose ratios between measurement and calculation for the head and neck plans were 1.000, 1.003, and 1.012 for the reference plan, ATP, and ATS, respectively.

#### 3.C.5 End‐to‐end testing

Data were transferred successfully between all systems, including the PET/CT scanner, TPS, and MOSAIQ, without any significant changes to institutional workflow. The agreement between the TPS and measured point doses for the CIRS end‐to‐end test was −0.01% and −0.34% for the ATP and ATS plans, respectively. The online dose calculation time for the ATP plan (adapt from segment weights) was 40 sec and 138 sec for the ATS plan.

The dose calculation time of the ATS for the IROC head and neck phantom was 910 sec. The axial and sagittal films within the phantom each had a gamma passing rate of 98% when evaluated with a 7% DD/4 mm DTA. All TLD point measurements were within 1% of the reported TPS value except for a single TLD located in the inferior and posterior region of the high‐dose PTV. This TLD had a 0.96 dose ratio between measurement and TPS dose calculation. Both the film and TLD measurements satisfy the IROC credentialing criteria. The measurement uncertainty associated with this phantom, as reported by IROC, is 3% for the TLD measurements and 1 mm for the spatial resolution of the film and densitometer.

## 4 Discussion

The Elekta Unity provides capabilities such as enhanced soft tissue contrast for tumor visualization and the ability to create online adaptive plans to account for daily anatomical variations. These capabilities will likely improve the safety and efficacy of treatment, but care must be taken to accurately commission this device for clinical use.

The results of the mechanical testing are within the recommended tolerances of AAPM TG‐142.^37^ The fixed‐ring design of the Unity allowed for a very tight isocenter with a diameter of magnitude 0.2 mm, which is tighter than the reported gantry isocenter diameters of standard linacs, including 1.0 mm for a Versa HD^21^ and 0.54 mm for a Varian Truebeam.^38^ Due to the large field size of the Unity and the extended source to imager distance of the EPID panel, it is only possible to acquire 30 of the 80 leaf pairs in a single MV image. Due to this limitation, film was utilized to verify the positional accuracy of all leaves. In the online adaptive workflow of the Unity, the patient cannot be shifted to isocenter, but rather the position of the leaves is changed through segment aperture morphing^6^ or re‐optimization and, therefore, leaf pairs farther off‐axis will likely be more heavily used than in standard linac designs and, thus, it was important to characterize their positional accuracy.

MRI commissioning was performed in accordance with the ACR and guidelines provided in the literature,^20^ but using sequences and techniques that are accessible to all users. As can be seen from the MRI results, the Marlin performs well in very close proximity to the linac components and gantry. In particular, the system is properly magnetically decoupled from all gantry components, including the linac with B_0_ maps showing a maximum peak‐to‐peak variation of 3.91 ppm or less over the full gantry rotation. The root mean square differences between each gantry angle tested and gantry angle 0° were found to be 0.10 ppm, although some variation in the homogeneity can be seen compared to gantry angle 0° (see Fig. 5). However, with these variations being small, they have not been found to have any impact clinically. The Marlin was also found to be decoupled from the radiofrequency noise of the linac, although some noise was seen to be introduced due to the motion of the MLC leaves. However, despite the noise introduced by the MLC, image degradation has not been observed.

As expected of an MRI that is to be used for treatment planning purposes, the Marlin shows good geometric accuracy at our center with 0.8 mm distortion over a 350 mm DSV and 1.1 mm at 400 mm. When planning to treat targets that are very peripheral in the field of view, these distortions should be considered. It was also noted that the gradient linearity shows a maximum error of less than 1 mm at a 400 mm DSV.

The image quality of the Marlin was of high quality as assessed by the ACR phantom, with all tests passing the recommended criteria. Image quality did degrade for imaging of moving targets using the clinical T2‐weighted navigator‐triggered sequence. Triggered imaging is currently the only technique available for patient setup imaging to manage respiratory motion. The navigator‐triggered imaging sequence provided was confirmed to capture images at the end‐expiration phase of the breathing cycle for both head first and feet first orientations. However, for high breathing rates of 20 bpm at total motions of 20 mm, it was discovered that the target could be misidentified by up to 3 mm based on the determination of the target contour centroid. Triggered imaging faithfully reproduced the target volume for all breathing rates tested to within 0.3 cm^3^, or within 0.20 mm diameter of a spherical target. As the average breathing rate of the irregular pattern was closer to 13–14 bpm, the volume and centroid for this test were close to the result of the periodic sinusoidal motions at 10 bpm. This shows that the navigator‐triggered sequence is not greatly affected by irregular breathing as tested. It is important to note that while the MR image acquisition is triggered, the radiation delivery cannot be in this release of the Unity. For this reason, care should be taken so as not to introduce a systematic offset between imaging and radiation delivery. A potential workflow that could incorporate navigator‐triggered imaging would be to create a reference plan on an exhale dataset and generate an ITV and PTV margins to account for the full range of motion and machine uncertainties. Online planning could then utilize the triggered image for daily registration to the reference plan exhale dataset and still have a PTV, which accounts for the full range of target motion.

The dosimetry of the system is complicated by the effects of the magnetic field and the nonuniform material in the beam path prior to reaching the patient, including the cryostat and couch. Effects such as the Lorentz force and electron return effect are handled by the GPU Monte Carlo dose calculation, and the TPS showed excellent agreement with measured profiles and PDDs. The cryostat attenuation as a function of gantry angle will vary from machine to machine largely from minor differences in construction such as welding. An attenuation map is entered into Monaco based off of the results from experimental measurement. The difference between measurement and TPS calculation of this attenuation map was within 1.2%. In addition to attenuation from the cryostat, the Unity couch is another source of significant attenuation. The couch can attenuate the beam by as much as 16.5% for beams passing through the highest‐density regions of the couch. Steep attenuation gradients are observed for couch angles between 110–135° and 230–255°. The agreement between measured and calculated attenuation for various gantry angles is shown in Table 5 where the discrepancy can be as high as 2.76%. Some of this discrepancy may be due to the steep gradient in output variation in regions of changing density within the couch. These results also show that improvement in the couch model may be warranted in future work, but in the current Monaco release, editing of the Unity couch model is disabled for users. In practice, most IMRT plans use multiple gantry angles, which will likely decrease the impact of these single‐beam angle differences.

The nonstandard geometry of the Unity, including the extended SSD, introduces additional complexity in the reference dosimetry calibration of the machine. In this work a beam specifier of $TPR_{10}^{20}$ was used and converted into a % dd(10)_x_ such that the remainder of the procedure would follow the AAPM TG‐51 guidelines. The measured $TPR_{10}^{20}$ was 0.704, which was similar to the value of 0.691 reported by O’Brien et al.^11^ The independent TLD validation was within 1% of our reported delivered dose, which supports this as a valid methodology for performing reference dosimetry. The total uncertainty of the reference dosimetry will closely mirror the value reported in the AAPM TG 51 addendum.^29^ TLDs were chosen as the dosimeter to perform independent validation as they do not have a statistically significant response dependence with and without magnetic fields and because they have minimal air gaps.^39^ However, the uncertainty of the TLDs, as reported by IROC, is 5%. This includes components such as calibration and readout. An additional uncertainty of approximately 0.5% based on the positioning of the TLDs can be expected from a 1 mm positional offset based on simulations from the TPS. Summing these uncertainties in quadrature yields a total uncertainty of approximately 5.0%.

All of the measured IMRT plans are within the recommended tolerances of AAPM TG‐218.^34^ Additionally, the adaptive IMRT quality assurance plans calculated based on a head and neck patient dataset had an average gamma pass rate of 99.7%, and point‐dose measurements were within 1% of calculation. End‐to‐end tests of the CIRS phantom for both adaptive workflows yielded measurements that were within 0.5% that of calculation. Although attempts were made to minimize air gaps in this setup, it is possible that small air gaps were still present, increasing the uncertainty of these measurements. Additionally, the film measurements and TLD point‐dose measurements of the MRgRT head and neck phantom meet the clinical trial credentialing criteria as specified by IROC. The online calculation of the MRgRT phantom was performed with a 2 mm dose grid which follows the recommendations of AAPM TG 101.^36^ The resulting calculation time with this dose grid was 910 sec which is likely too long to be utilized clinically. SBRT dose calculations will likely require a larger dose grid than recommended to achieve feasible online dose re‐optimizations which illustrates a limitation of the Unity in its current state. In general, the dose calculations show a high degree of accuracy between the TPS and measurement for reference plans and adaptive planning, where challenges in performing patient‐specific QA add even more importance to an accurate primary dose calculation.

Some limitations of this study include the use of vendor supplied software and phantoms for specific tests. While this may not be ideal, third‐party vendor phantoms and software for many of the tests described in this work are not commercially available, which highlights a need within the field. This work outlines possible methodologies using currently available software and phantoms in the commissioning process of the Unity. We acknowledge that there are other possible techniques that are currently available and in development such as novel QA software and 3D end‐to‐end phantoms^40^ which may be alternatively utilized.

## 5 Conclusion

Mechanical testing, MRI commissioning, and dosimetric validation were performed in accordance with appropriate task group reports and vendor recommendations. Aspects of the MRI that are important for adaptive planning have been commissioned, and baseline values have been obtained. The mechanical components are within recommended tolerances, and an independent end‐to‐end dosimetric validation was performed on a head and neck phantom, which met all IROC criteria.

## Conflict of Interest

All authors declare no conflicts of interest relevant to this work.
